# The chiropractic workforce: a global review

**DOI:** 10.1186/s12998-019-0255-x

**Published:** 2019-07-24

**Authors:** Mette Jensen Stochkendahl, Mana Rezai, Paola Torres, Deborah Sutton, Peter Tuchin, Richard Brown, Pierre Côté

**Affiliations:** 1 Nordic Institute of Chiropractic and Clinical Biomechanics, Odense, Denmark; 2 Department of Sports Science and Clinical Biomechanics, University of Southern Denmark, Campusvej 55, DK-5230 Odense M, Denmark; 3 UOIT-CMCC Centre for Disability Prevention and Rehabilitation, 2000 Simcoe St N, Oshawa, ON L1G 0C5 Canada; 4 Exercise Science Laboratory, School of Kinesiology, Faculty of Medicine, Universidad Finis Terrae, Avda Pedro de Valdivia 1509, Providencia, Santiago, Chile; 5 Department of Chiropractic, Faculty of Science and Engineering, Room 342, Bldg. C5C West, Sydney, NSW 2109 Australia; 6 World Federation of Chiropractic, 160 Eglinton Avenue East (#601), Toronto, Ontario M4P 3B5 Canada; 7 Faculty of Health Sciences, University of Ontario Institute of Technology, Oshawa, Canada; 8 Dalla Lana School of Public Health, University of Toronto, 155 College St Room 500, Toronto, ON M5T 3M7 Canada

**Keywords:** Chiropractic, Rehabilitation, Global Health, Health workforce, Survey, Cross-sectional, Education

## Abstract

**Background:**

The world is faced with a chronic shortage of health workers, and the World Health Organization (WHO) has estimated a global shortage of 7.2 million health workers resulting in large gaps in service provision for people with disability. The magnitude of the unmet needs, especially within musculoskeletal conditions, is not well established as global data on health work resources are scarce.

**Methods:**

We conducted an international, cross-sectional survey of all 193 United Nation member countries and seven dependencies to describe the global chiropractic workforce in terms of the *availability* (numbers and where they are practising), *quality* (education and licensing), *accessibility* (entry and reimbursement), and *acceptability* (scope of practice and legal rights). An electronic survey was issued to contact persons of constituent member associations of the World Federation of Chiropractic (WFC). In addition, data were collected from government websites, personal communication and internet searches. Data were analysed using descriptive statistics. Worldwide density maps of the distribution of numbers of chiropractors and providers of chiropractic education were graphically presented.

**Results:**

Information was available from 90 countries in which at least one chiropractor was present. The total number of chiropractors worldwide was 103,469. The number of chiropractors per country ranged from 1 to 77,000 (median = 10; IQR = [4–113]). Chiropractic education was offered in 48 institutions in 19 countries. Direct access to chiropractic services was available in 81 (90%) countries, and services were partially or fully covered by government and/or private health schemes in 46 (51.1%) countries. The practice of chiropractic was legally recognized in 68 (75.6%) of the 90 countries. It was explicitly illegal in 12 (13.3%) countries.

**Conclusion:**

We have provided information about the global chiropractic workforce. The profession is represented in 90 countries, but the distribution of chiropractors and chiropractic educational institutions, and governing legislations and regulations largely favour high-income countries. There is a large under-representation in low- and middle-income countries in terms of provision of services, education and legislative and regulatory frameworks, and the available data from these countries are limited.

**Electronic supplementary material:**

The online version of this article (10.1186/s12998-019-0255-x) contains supplementary material, which is available to authorized users.

## Background

Health workers are the engine of our health care systems. Yet, the world is faced with a chronic shortage of health workers. In 2013, the World Health Organization (WHO) estimated a global shortage of 7.2 million health workers, with 83 countries facing a health worker crisis [1].

In the twenty-first century, the world faces a new set of health challenges: a rapid increase in the number of care-seeking elderly people in the population accompanied by a rise in chronic conditions and multi-morbidity [2]. According to the World Health Survey [3], which investigated adults in 59 countries, the average prevalence of disability ranged from 11.8% in high income countries to 18.0% in low income countries. This equates to an estimated figure of more than one billion people. Musculoskeletal pain is the main cause of disability and the prevalence and burden increase with age [4]. Musculoskeletal pain causes more years lived with disability than any other condition creating a substantial and increasing unmet need for health care and rehabilitation services [4–6]. Health systems must face these emerging challenges by developing health policies that emphasize health care services that improve functioning [7].

In 2006, WHO established the Global Health Workforce Alliance as a common platform for action to address issues of health workforce shortage. At the end of the Alliance’s ten-year mandate, the Global Health Workforce Network [8] was established to continue the Alliance’s work by informing and maintaining high-level political engagement and support the implementation of the Global Strategy on Human Resources for Health: Workforce 2030 [9]. The Global Strategy on Human Resources for Health: Workforce 2030 aims to ensure universal *availability, accessibility, acceptability, coverage and quality* of health workforces within strengthened health systems (World Health Assembly 2016) [10]. *Availability* is the sufficient supply and stock of health workers, with the relevant competencies and skill mix that correspond to the health needs of the population. *Accessibility* relates to equitable access to health workers (including travel time and transport, opening hours and corresponding workforce attendance) in a disability-friendly infrastructure with referral mechanisms in place. *Acceptability* relates to the characteristics and ability of the workforce to treat everyone with dignity, create trust and enable or promote demand for services. *Quality* refers to the competencies, skills, knowledge and behaviour of the health worker as assessed according to professional norms and as perceived by users [1].

To support the implementation of the Global Strategy on Human Resources for Health: Workforce 2030, the National Health Workforce Accounts: A Handbook (NHWA) was developed to assist researchers and policy makers to describe and evaluate health workforces [10]. The underpinning framework of NHWA is aligned with the health labour market framework. It provides a comprehensive picture of the education sector and health labour market dynamics, in which the economy, population, politics and society act as drivers to attaining universal health coverage and optimal health-care services as the outcome [10]. The NHWA contains 78 core indicators divided into 10 modules which measure three crucial labour market components: education (i.e. the “input” of health workforces), labour force (i.e. the “throughput”) and serving population health needs (i.e. the “output”).

Rehabilitation as provided by chiropractors, physiotherapists and similar allied health professionals has a critical role to play in preventing and minimizing the impact of musculoskeletal conditions on associated limitations in functioning (e.g. in mobility, activities of work and daily life) and chronic disability [7]. Addressing people’s limitations in functioning may help to minimize the health, social and economic impacts of health conditions, and improve well-being. The availability of accessible and affordable rehabilitation is necessary for many individuals to remain independent, participate in education, be economically productive, and fulfil meaningful life roles within their communities. However, global data on availability are scarce. Initial findings reveal large gaps in service provision for people with disability. For example, in Zimbabwe, Malawi, Zambia and Namibia, 60 to 68% of people with disabilities expressed an unmet need for medical rehabilitation [3]. Similarly, the geographical location and actual number of chiropractors worldwide is unknown, and their characteristics only partially described. Further, gender inequality co-exists with higher unmet needs in women as well as a reduced service provision in rural areas. The magnitude and scope of unmet rehabilitation needs, especially in low- and middle-income countries requires a concerted and coordinated global action by all stakeholders [7].

## Aims

We aimed to describe the chiropractic workforce worldwide in terms of the number of chiropractors, education, access, reimbursement schemes, scope of practice, and legal rights.

Using the WHO NHWA, we asked:

1. What is the global *availability* of chiropractors and where are they practising?
2. What is the *quality* of the chiropractic workforce (including education, postgraduate education, and licensing)?
3. What is the *accessibility* to chiropractic services (entry and reimbursement)?
4. What is the global *acceptability* of chiropractors (i.e. what is the scope of practice and legal rights of the chiropractic profession)?

## Methods

We conducted an international, cross-sectional survey of all 193 United Nation (UN) member countries and seven dependencies (hereafter called countries). Based on lists of contact persons of constituent member associations of the World Federation of Chiropractic (WFC), one contact in each country was invited to participate on behalf of their country. Where the WFC had no contacts, data were collected from government websites or from chiropractors practising in the country found through web searches using the Google search engine. The designated contacts of the WFC were invited to participate via email in September 2016. A link in the email enabled the participant to directly access the survey using an online survey tool, *Survey Monkey*. A re-writable pdf copy was also available for direct email response. Two email reminders were sent to enhance the response rate (two and 4 months after the initial invitation). Further, the survey was resent to non-responders in February 2017, and subsequently, the WFC administrative team and Council members made direct contact with contacts in their regions to further encourage participation in the survey. Partial responders were prompted via email to complete requested information.

### The survey

The instrument (Additional file 1) draws on previous, regular surveys of the legal status of chiropractic in WFC constituent member associations’ jurisdictions designed and posted triennially by the WFC. It included 38 questions inquiring about the: 1) legal status of chiropractic (*n* = 15); 2) scope of practice (*n* = 6); 3) privileges to order imaging, laboratory tests and prescribe drug prescription (*n* = 5); 4) regulations regarding care of children (*n* = 1); 5) sickness absence certification rights (*n* = 1); 6) reimbursement schemes (*n* = 3); 7) education (*n* = 5); and 8) statutory requirements of practice for foreign trained chiropractors (*n* = 2). Response categories were predominantly dichotomous (yes/no), categorical or multiple choice. Throughout the questionnaire, respondents had the opportunity to elaborate on answers as free text. Before releasing the survey, the questions were assessed for face and content validity by one of the WFC constituent member representatives and adapted accordingly.

### Validation of data and missing data

Where possible, responses (such as legislation and number of registered DCs in a country) were systematically checked against posted data in the relevant government websites to obtain current data. If the website had no posted data, the relevant national health authority website was consulted for further information. If data were missing, data from WFC records from previous surveys were imputed when available. Emails were sent to WFC contacts to confirm the imputed data.

### Data analysis

Data were analysed using descriptive statistics. Dichotomous and multi-choice variables were described using frequencies and proportions. Continuous data were described as raw numbers and as medians and interquartile ranges (IQR). Worldwide density maps of the distribution of numbers of chiropractors and chiropractic educations are graphically presented.

## Results

**Fig. 1:**
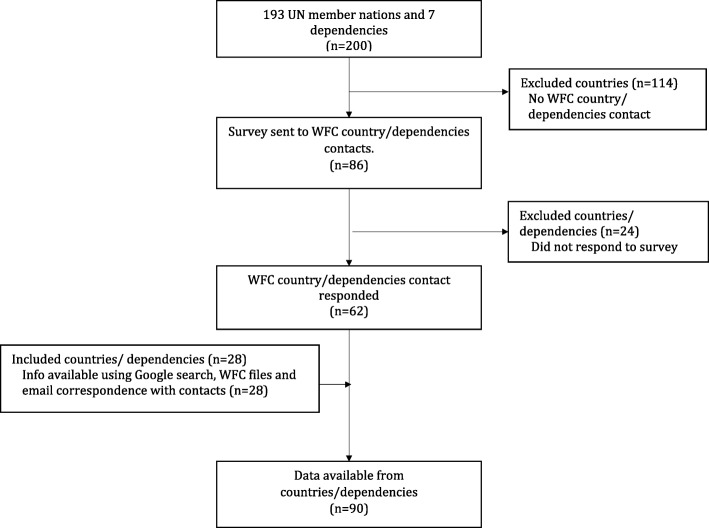
Flowchart – included and excluded countries and dependencies

Questions were issued to 86 country contacts. Sixty-two contacts responded (response rate of 72%). Further information was collected from WFC files, email correspondence with WFC contacts, and/or Google searches in 28 countries (Fig. 1). In total, information was available in 90 countries; 27 countries in Europe, 22 in Asia, 16 in Africa, 9 in North America and the Caribbean, 12 in South America, and 4 in Australia and the Pacific.

### Availability of chiropractors

In all 90 countries, at least one chiropractor was present. The total number of chiropractors worldwide was 103,469. The number of chiropractors per country ranged from one to 77,000 (median = 10; IQR = [4–113]). The median chiropractor per capita per 100,000 was 0.26 (IQR = [0.028–1.40]).

**Fig. 2:**
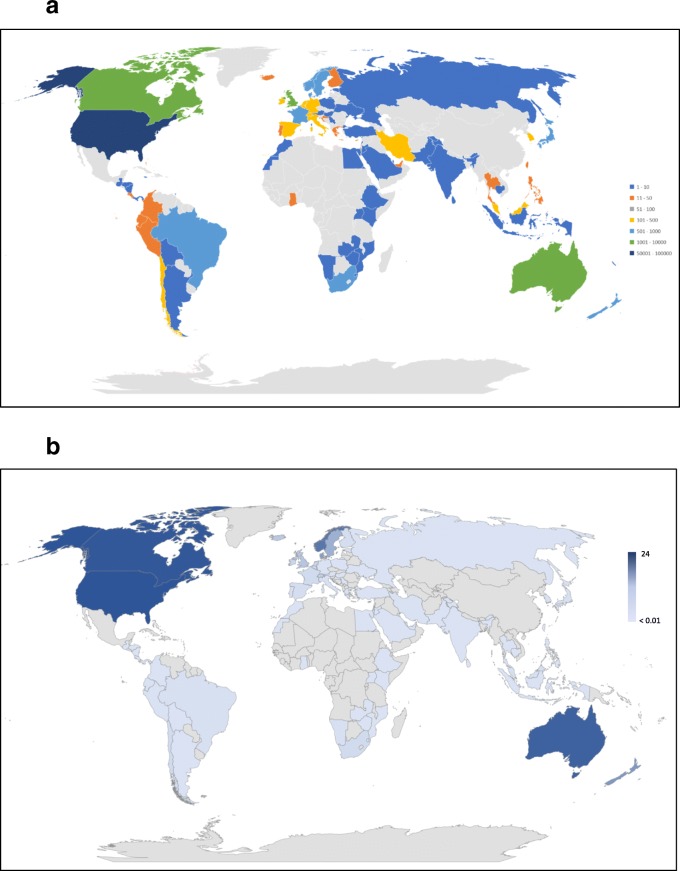
**a** Countries by population of chiropractors in absolute numbers. **b** Countries by population of chiropractors per capita per 100,000

The US had the highest number of chiropractors per capita; for every 100,000 people in the US, there were 23.7 chiropractors. India had the lowest number of chiropractors per capita; for every 100,000 people in India, there were 0.0007 chiropractors. Density maps of the absolute number of chiropractors worldwide and per capita per 100,000 are presented in Fig. 2a and b.

### Quality of the chiropractic workforce

**Fig. 3:**
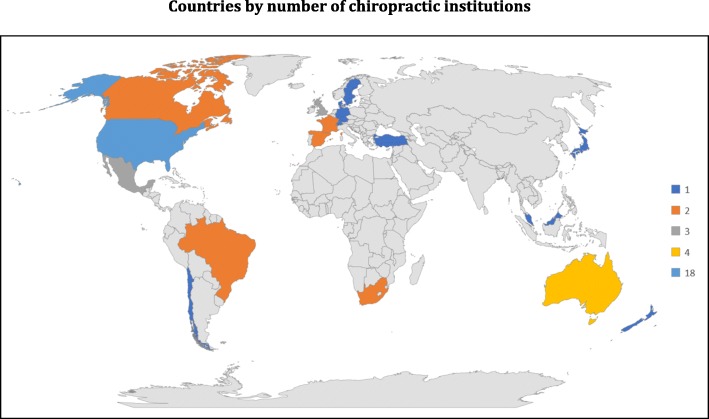
Countries by number of chiropractic institutions

Chiropractic education was offered in 48 institutions in 19 countries. They were predominantly located in English-speaking and/or high-income countries. The number of chiropractic educational institutions in each country ranged from 1 to 18 (Fig. 3) with the majority located in the US (*n* = 18) and Australia (*n* = 4). Ten countries had only one educational institution. In 16 of the 18 countries with an educational institution, the education was accredited by an international chiropractic accreditation counsel, government/federal institution or both. Further details about institutional accreditation and requirements for practice are provided in Table 1.

**Table 1:** Chiropractic education and requirements for practice (*N* = 90)

	n	(%)
Chiropractic education institutions		
Is chiropractic education provided in your country?		
Yes	19	(23.5)
No	58	(64.4)
Missing	14	(15.6)
Accreditation of the chiropractic educational institutions^a^		
By an international chiropractic accreditation counsel	11	(12.2)
By a governmental/federal institution	5	(5.6)
By both	11	(12.2)
Missing	74	(82.2)
The level of chiropractic training is provided by law		
Yes	25	(27.8)
No	36	(40.0)
Missing	29	(32.2)
Requirements to practice		
Is completion of a post-graduate clinical training program required to obtain full licensure or registration to practice as a chiropractor		
Yes (1 or 2 years)	15	(16.7)
No	39	(43.3)
Missing	36	(40.0)
For overseas chiropractors wishing to practice chiropractic in your country, please indicate below which statutory requirements apply^a^		
Work permit	59	(65.6)
Visa	45	(50.0)
Registration with statutory regulator	36	(40.0)
Evidence of health	18	(20.0)
Language proficiency	23	(25.6)
Malpractice insurance	24	(26.7)
Certification of good standing	27	(30.0)
Other (e.g. proof of qualifications, board exams)	19	(21.1)
Overseas chiropractors not permitted to work	2	(2.2)
Missing	23	(25.6)

^a^This may add up to more than 100%, as multiple answers may apply

### Accessibility to chiropractic services

Direct access to chiropractic services was available in 81 (90%) countries, and medical referral was needed in only two countries, the Bahamas and Saudi Arabia. Chiropractic services were partially or fully covered by government and/or private health schemes in 46 (51.1%) countries. Details are provided in Table 2.

**Table 2:** Accessibility to chiropractic services and scope of practice (*N* = 90)

	n	(%)
Access and reimbursement		
Public access to chiropractors		
Direct access	80	(88.9)
Direct access under supervision of medical doctor	1	(1.1)
Medical referral needed	2	(2.2)
Missing	7	(7.8)
Are chiropractors’ fees covered by government health schemes?		
Yes	16	(17.8)
No	45	(50.0)
Missing	29	(32.2)
Are chiropractors’ fees covered by private health schemes?		
Yes, all major schemes	18	(20.0)
Yes, some major schemes	28	(31.1)
No	15	(16.7)
Missing	29	(32.2)
Definition of chiropractic		
Does the legislation include a definition of chiropractic?		
Yes	25	(27.8)
No	25	(27.8)
Missing	40	(44.4)
Is scope of practice defined in law or regulations?		
Yes	26	(28.9)
No	26	(28.9)
Missing	38	(42.2)
Scope of practice: are chiropractors lawfully permitted to undertake the following activities^a^		
Myofascial dry needling/acupuncture	20	(22.2)
Prescription of food supplements	24	(26.7)
Prescription of herbal remedies	19	(21.1)
Laser therapy	23	(25.6)
Ultrasound/interferential/shockwave/other electrical therapy	29	(32.2)
Missing	54	(60.0)
Are prescribed acts included in the legislation?		
Yes	9	(10.0)
No	20	(22.2)
Missing	61	(67.8)
Does the legislation confer protection of title?		
Yes	39	(43.3)
No	11	(12.2)
Missing	49	(44.4)
Diagnostic rights		
In relation to plain film x-rays, are chiropractors lawfully permitted^a^		
To own and operate x-ray equipment within their clinics	13	(14.4)
To prescribe/order x-rays (using an outside provider) within the state health system	28	(31.1)
To prescribe/order x-rays (using an outside provider) privately	35	(38.9)
Missing	47	(52.2)
In relation to advanced diagnostic imaging chiropractors lawfully are permitted		
To prescribe/order advanced MRI/CT scans	22	(24.4)
Missing	68	(75.6)
In relation to diagnostic ultrasound, are chiropractors lawfully permitted^a^:		
To own and operate ultrasound equipment within their clinics	14	(15.6)
To prescribe/order ultrasound studies (using an outside provider)	32	(35.6)
Missing	56	(62.2)
In relation to laboratory tests (blood, urine, etc.), are chiropractors lawfully permitted		
To prescribe/order laboratory tests	29	(32.2)
No rights	30	(33.3)
Missing	31	(34.4)
Treatment and prescription rights		
Are/do chiropractors lawfully permitted/have statutory rights in relation to the prescription of pharmaceutical medication?		
Full prescribing rights without additional training	1	(1.1)
Full prescribing rights with additional training	0	–
Limited prescribing rights without additional training	4	(4.4)
Limited prescribing rights with additional training	4	(4.4)
No prescribing rights	57	(63.3)
Missing	24	(26.7)
Are chiropractors subject to any specific regulations/statutory restrictions in relation to the care of children?		
Yes	57	(63.3)
No	3	(3.3)
Missing	30	(33.3)
Are chiropractors lawfully permitted to authorize sick leave?		
Yes	20	(22.2)
No	40	(44.4)
Missing	30	(33.3)

^a^This may add up to more than 100%, as multiple answers may apply

### Acceptability of chiropractors

The scope of chiropractic practice was governed by legislation or regulation in 26 (28.9%) countries and the professional title protected by legislation in 39 (43.3%) (Table 2). In 43 (47.8%) countries, chiropractors are permitted to own, operate, or prescribe x-rays, in 22 (24.4%) countries they were lawfully permitted to prescribe advanced imaging (MRI or CT), and in 34 (38.8%) countries owning, operating or prescribing diagnostic ultrasound was permitted. Full or limited rights to the prescription of pharmaceutical medication were permitted in 9 (10%) countries, and authorization of sick leave was permitted in 20 (22.2%) countries. The care of children is subject to specific regulations and/or statutory restrictions in 57 (63.3%) countries.

The practice of chiropractic was legally recognized in 68 (75.6%) of the 90 countries. It was explicitly illegal in 12 (13.3%) countries; one in Africa (Egypt), two in South America (Argentina and Columbia), four in Europe (Austria, Estonia, Greece, and Hungary), and five in Asia (Lebanon, Republic of Korea, Taiwan, Turkey and Ukraine). In the remaining 10 (11.1%) countries, chiropractic did not fall under any law or the statutory legislation is unknown. The characteristics of the statutory legislation regarding chiropractic in the 68 countries in which chiropractic was legalized are provided in Table 3. In the majority of the 68 countries (*n* = 48, (70.6%)), chiropractic was governed by a legal framework under national/federal or regional/state legislation or a combination, and in 44 countries (64.7%), the legislation contains provision to establish a statutory regulator. Forty-two countries (61.8%) have regulations and/or rules under the legislation to provide for registration or licensure of chiropractors.

**Table 3:** Statutory legislation in countries where chiropractic is legalized (*N* = 68)

	n	(%)
The legal framework governing chiropractic is		
National/Federal legislation (i.e. covers the entire country)	43	(63.2)
Regional/State/Provincial/Canton legislation	2	(2.9)
A combination of national/federal legislation with regional/state/provincial/canton rules	3	(4.4)
Missing	20	(29.4)
The chiropractic legislation stands alone		
Yes	13	(19.1)
No	55	(80.9)
Missing	0	–
The legislation contains provision to establish a statutory regulator		
Yes	44	(64.7)
No	6	(8.8)
Missing	18	(26.5)
A statutory regulator exists to set standards		
Yes	27	(39.7)
Missing	41	(60.3)
Roles of the statutory regulator^a^		
Maintain a statutory register	22	(32.4)
Set standards of ethics/conduct and proficiency	22	(32.4)
Set standards of education	14	(20.6)
Accredit educational programs	12	(17.7)
Set standards of continuing education	11	(16.2)
Set standards of health	11	(16.2)
Administer fitness to practice (disciplinary and health) hearings and impose sanctions	18	(26.5)
Missing	41	(60.3)
Regulations and/or rules under the legislation to provide for registration or licensure of chiropractors		
National/federal level.	39	(57.4)
State/provincial level.	3	(4.4)
None	8	(11.8)
Missing	18	(26.5)
The legal framework contains provision for disciplinary/fitness to practise hearings		
Yes	31	(45.6)
No	10	(14.7)
Missing	27	(39.7)
Practicing without a license/without being registered is an offence		
Criminal offence	13	(19.1)
Civil offence	15	(22.1)
May practice but cannot use title	1	(1.5)
Missing	39	(57.4)
Sanctions available when a chiropractor has been found guilty of unacceptable professional conduct/serious professional misconduct under the legal framework^a^		
Removal from the register (striking off)	23	(12.0)
Admonishment	21	(11.0)
Written undertakings	10	(5.0)
Conditions of practice order	12	(6.0)
Suspension from practice	21	(11.0)
Financial penalty/fine	11	(6.0)
Other	2	(1.0)
Missing	42	(61.8)

^a^This may add up to more than 100%, as multiple answers may apply

## Discussion

We have surveyed the chiropractic workforce in all UN member countries using an electronic survey of contact persons of constituent member associations of the WFC, WFC files, personal correspondence, government websites, and Google searches. Chiropractors are available in 90 countries, but 50% percent of these have 10 or fewer chiropractors. The lowest provision of chiropractic services, both absolutely and per capita, is found in low- and middle-income countries. This is reflective of the location of chiropractic educational institutions in few, predominantly high-income countries. These institutions are accredited by government/federal institutions or international chiropractic accreditation counsels, which warrant a standard level of quality. But, many receive little or no public financial support, so access to education is for the few who can afford tuition fees. Direct access to chiropractic services is ensured in most countries, but public reimbursement schemes are few, which again limits the access to those who can afford either the service fee or private health insurance payment. Finally, we have observed variation in scope of practice. Importantly, the care of children is under regulation or restrictions in most countries, and chiropractors are only legally recognized in 68 of the 90 countries, and explicitly illegal in 12 countries.

In comparison to similar rehabilitaton professions such as physiotherapy, the chiropractic workforce is relatively small. This may partly be due to the education of physiotherapists, which is often incorportated in the faculty of medicine or rehabilitiation sciences of major univerities as opposed to the chiropractic education, which is predominantly offered at independent schools or colleges, at least in North America. Regardless, the variations in the density of the chiropractic workforce both within and across high-, middle-, and low-income countries are mirrored by other professions. The World Confederation of Physical Therapy reports an estimated number of 450,000 physical therapists worldwide with the number of physio therapists per capita per 100,000 ranging from 0.19 in Malawi to 282 in Finland [11]. When comparing to other rehabilitation providers, in the United States there are 13.8–25.4 occupational therapists for every 100,000 people and 27.3–37.2 speech language pathologists for every 100,000 people [12].

We used multiple resources to get the best possible overview of the chiropractic workforce. The number of chiropractors is a crude estimate based on membership of national or regional associations, but not all chiropractors are members of an association, and some may be members of more than one. Globally, central national registers of the human health workforce are the exception rather than the rule, and the information of rehabilitation staff is fragmented and insufficient [13]. This has been attributed to the lack of common definitions, inadequate resources needed to monitor the workforce, and little or no political determination [13].

The main data collection method was the electronic survey, but we also gathered data from web searches, WFC records and personal communication to supplement, validate and increase the granularity of the data. Despite this, we have a large proportion of missing data, and we do not know the validity or accuracy of the data collected in the survey. Responders to the survey were predominantly from countries with well-established, larger cohorts of chiropractors organized in national or regional associations, well-defined legislation, and the presence of a chiropractic educational institution. This limits the findings of this report, but is, however, reflective of the challenges associated with the of lack of chiropractic resources in many countries. From the personal communication with WFC contacts, lack of or poor internet connections, unfamiliarity with surveys, and legislative struggles in some countries, or the lack of applicability of questions in countries with unclear legislation or regulation were reported as barriers for responding. Similarly, missing data limit the generalizability of results. Finally, the survey was issued in English only, which may have led to misinterpretations of questions, or deterred some from responding to parts of or the whole survey.

In this report, we described the characteristics of the chiropractic workforce worldwide to improve our global understanding of rehabilitation resources. This report highlights the availability of chiropractic educational institutions, post-graduate training, accreditation, the active health workforce stock, governance, and health workforce policies. To fully map the chiropractic workforce in accordance to the NHWA, more information is still needed on education finances, health labour market flow, employment characteristics and working conditions, health workforce spending and remunerations, skill-mix composition for models of care, and health workforce information systems. Further, issues such as gender and ethnic inequality and underserved communities need to be investigated. Due to the large variation in numbers, legislation and scope of practice, this may be best done at national levels and perhaps undertaken alongside investigations of other rehabilitation professions, such as physiotherapy and other types of manual and exercise therapists.

## Conclusion

In accordance with the WHO NHWA framework, we have provided information about the global chiropractic workforce in terms of the availability, quality, accessibility, and acceptability. The profession is represented in 90 countries, but the distribution, chiropractic educational institutions, and governing legislations and regulations largely favour high-income countries. There is a large under-representation in low- and middle-income countries in terms of provision of services, education and legislative and regulatory frameworks, and the available data from these countries are limited.

## Additional file

Additional file 1: Survey questions and response options. (DOCX 19 kb)

## Abbreviations

EU GDPR: European Union General Data Protection Regulation
IQR: Inter Quartile Range
NHWA: National Health Workforce Accounts
UN: United Nations
WFC: World Federation of Chiropracic
WHO: World Health Organization

## Authors’ information

The authors are members of the World Federation of Chiropractic Disability and Rehabilitation Committee (WFC-DRC) and the presented work was undertaken on a mandate of WFC-DRC. The committee was formed by the World Federation of Chiropractic in response to a recognized need for expert input to inform its work.
