# Supplementary material for: The chiropractic workforce: a global review
Source: Chiropr Man Therap. 2019 Jul 24;27:36. doi: 10.1186/s12998-019-0255-x (PMC6651973; doi:10.1186/s12998-019-0255-x)
Supplement: Supplementary file 1 — Survey questions and response options. (DOCX 19 kb) [file 12998_2019_255_MOESM1_ESM.docx]

**Additional file 1 - Survey questions and response options**

| **Item** | **Question** | **Response option** |
| --- | --- | --- |
|  | Legal information (n=2) |  |
| 1 | Is the practice of chiropractic legally recognized in your country? | Yes/no |
| 2 | Where there is no dedicated legislation, is the practice of chiropractic: | Lawful under common law  Technically illegal but not enforced by criminal prosecution  Explicitly illegal and subject to prosecution |
|  | WITH legislation (n=) |  |
| 3 | In relation to the legal framework governing chiropractic, is it: | National/Federal legislation (i.e. covers the entire country)  Regional/State/Provincial/Canton legislation  A combination of national/federal legislation with regional/state/provincial/canton rules |
| 4 | Is the chiropractic legislation standalone (i.e. a dedicated Chiropractic Act)? | Yes  No (legislation is incorporated into general provisions for health professionals) |
| 5 | In what year was legislation first passed? | integer |
| 6 | Please provide the website link/s to the legislation. | free text |
| 7 | Does the legislation contain provision to establish statutory regulator? | Yes/no |
| 8 | Does a statutory regulator exist to set standards? | Yes/no |
|  | What are the roles of the statutory regulator? | Maintain a statutory register  Set standards of ethics/conduct and proficiency  Set standards of education  Accredit educational programs  Set standards of continuing education  Set standards of health  Administer fitness to practice (disciplinary and health) hearings and impose sanctions |
| 9 | Does the legal framework (legislation, rules, regulations, etc) permit direct access to chiropractors without medical referral? | Yes/no |
| 10 | Please list the regulatory body/bodies and provide their website links. | Free text |
| 11 | Are there regulations and/or rules under the legislation to provide for registration or licensure of chiropractors? | Yes, national/federal.  Yes, state/provincial.  No  In progress |
| 12 | Under legislation, how many registered/ licensed chiropractors are there in your country? | integer |
| 13 | Does the legal framework contain provision for disciplinary/fitness to practise hearings? | Yes/no |
| 14 | Is practising without a license/without being registered an offence? | Yes, criminal offence  Yes, civil offence  No |
| 15 | Under the legal framework, what sanctions are available when a chiropractor has been found guilty of unacceptable professional conduct/serious professional misconduct? | Warning  Admonishment  Written undertakings  Conditions of practice order  Suspension from practice  Removal from the register (striking off)  Financial penalty/fine  Other (please detail) |
|  | Scope of practice |  |
| 16 | Does the legislation include a definition of chiropractic? | No  Yes |
| 17 | List relevant definition/s where possible in English. If English is not used in your legislation, please submit in your native language the definition used. | Free text |
| 18 | Is scope of practice defined in law or regulations? | Yes  No  Additional information |
| 19 | Scope of practice: are chiropractors lawfully permitted to undertake the following activities: | Myofascial dry needling/acupuncture  Food supplement prescription  Herbal remedy prescription  Laser therapy  Ultrasound/interferential/shockwave/other electrical therapy |
| 20 | Are prescribed acts included in the legislation? | Yes/no - please provide details |
| 21 | Does the legislation confer protection of title?  *(E.g. provisions saying that only registered/ licensed chiropractors may use the title chiropractor or otherwise hold themselves out as practicing chiropractic)*  *Authorized titles may include chiropractor, Doctor of Chiropractic, chiropractic physician, chiropractitioner* | Yes/no  Further comment (if necessary) |
|  | Imaging rights |  |
| 22 | A. In relation to plain film x-rays, are chiropractors lawfully permitted: | To own and operate x-ray equipment within their clinics  To prescribe/order x-rays (using an outside provider) within the state health system  To prescribe/order x-rays (using an outside provider) privately  No rights  other |
| 23 | In relation to advanced diagnostic imaging (CT, MRI, etc.), are chiropractors lawfully permitted: | To prescribe/order advanced diagnostic imaging within the state health system  To prescribe/order advanced diagnostic imaging privately  All of the above  No rights  Other (please specify) |
| 24 | In relation to diagnostic ultrasound, are chiropractors lawfully permitted: | To own and operate ultrasound equipment within their clinics  To prescribe/order ultrasound studies (using an outside provider) within the state health system  To prescribe/order ultrasound studies (using an outside provider) privately  No rights |
|  | Drug prescription |  |
| 25 | Are/do chiropractors lawfully permitted/have statutory rights in relation to the prescription of pharmaceutical medication? | Full prescribing rights without additional training  Full prescribing rights with additional training  Limited prescribing rights without additional training  Limited prescribing rights with additional training  No prescribing rights  Please add additional comments |
|  | Laboratory tests |  |
| 26 | In relation to laboratory tests (blood, urine, etc.), are chiropractors lawfully permitted: | To prescribe/order laboratory tests within the state health system  To prescribe/order laboratory tests privately  All of the above  No rights  Other (please specify) |
|  | Paediatric care |  |
| 27 | Are chiropractors subject to any specific regulations/statutory restrictions in relation to the care of children? | Yes (please provide the details below)  No |
|  | Sick leave/time off work | Yes/no |
| 28 | Are chiropractors lawfully permitted to authorize sick leave? |  |
|  | Reimbursement |  |
| 29 | Are chiropractors’ fees covered by government health schemes? | Yes (please detail any specific conditions below)  No |
| 30 | Are chiropractors’ fees covered by private health schemes? | All major schemes (please detail any specific conditions below)  Some major schemes (please detail any specific conditions below)  No coverage |
| 31 | Please estimate the percentage of chiropractic fees that are reimbursed, whether by government or privately | 0%  1-20%  21-50%  51-74%  75-99% |
|  | Requirements to practice chiropractic in your country |  |
| 32 | For overseas chiropractors wishing to practice chiropractic in your country, please indicate below which statutory requirements apply. | 1. Work permit  2. Visa  3. Registration with the statutory/regulatory/state board  4. Evidence of current health status  5. Evidence of language proficiency  6. Malpractice insurance  7. Certificate/evidence of good standing from previous jurisdiction  8. Other (Please specify) |
| 33 | Provide the corresponding website links for selected responses above | Free text |
|  | Education |  |
| 34 | Is chiropractic education provided in your country? | Yes/no |
| 35 | If yes, how many educational institutions are there in your country? | integer |
| 36 | Chiropractic educational institutions in your country are: | Nationally/federally recognized Recognized/approved by national chiropractic association  Recognized by both  Not recognized |
| 37 | Is the level of chiropractic education prescribed by law? | Yes  No |
| 38 | Is completion of a post-graduate clinical training program required to obtain full licensure or registration to  practice as a chiropractor in your country? | Not required.  Yes. (Please complete details below e.g. duration, qualification, etc.) |
